# Adult-type and Pediatric-type Diffuse Gliomas: What the Neuroradiologist Should Know

**DOI:** 10.1007/s00062-023-01277-z

**Published:** 2023-03-20

**Authors:** Reinhold Nafe, Luciana Porto, Patrick-Felix Samp, Se-Jong You, Elke Hattingen

**Affiliations:** Dept. Neuroradiology, Clinics of Johann Wolfgang-Goethe University, Schleusenweg 2–16, 60528 Frankfurt am Main, Germany

**Keywords:** Astrocytomas, Oligodendrogliomas, Glioblastomas, Diffuse midline gliomas, Diffuse hemispheric gliomas

## Abstract

The classification of diffuse gliomas into the adult type and the pediatric type is the new basis for the diagnosis and clinical evaluation. The knowledge for the neuroradiologist should not remain limited to radiological aspects but should be based additionally on the current edition of the World Health Organization (WHO) classification of tumors of the central nervous system (CNS). This classification defines the 11 entities of diffuse gliomas, which are included in the 3 large groups of adult-type diffuse gliomas, pediatric-type diffuse low-grade gliomas, and pediatric-type diffuse high-grade gliomas. This article provides a detailed overview of important molecular, morphological, and clinical aspects for all 11 entities, such as typical genetic alterations, age distribution, variability of the tumor localization, variability of histopathological and radiological findings within each entity, as well as currently available statistical information on prognosis and outcome. Important differential diagnoses are also discussed.

## Introduction

Molecular alterations have become the most important diagnostic criteria for classification of tumor entities over the last 10 years. The current classification of diffuse gliomas is an appropriate example for the fact that the molecular profile is nowadays the most important biomarker to assess the biological behavior of a tumor besides morphological criteria from histopathology, immunohistochemistry, and radiological imaging. This is a fundamental new aspect in routine diagnostics, as many tumors, which do not show morphological features of a higher tumor grade, are classified as high-grade tumors based on molecular analysis. It is important to be aware of currently published interdisciplinary diagnostic experience with the individual tumor entities of diffuse gliomas to assess the diagnostic significance of morphologic findings and of additional diagnostic imaging techniques in individual cases. As an important point, tumor grades are now written in Arabic instead of Roman numerals.

## Adult-type Diffuse Gliomas

**Fig. 1:**
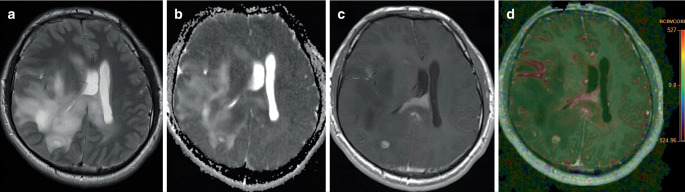
**a**–**d** Diffuse astrocytoma with mutation of isocitrate dehydrogenase (IDH-mutation) and cyclin dependent kinase 2A homozygous deletion (CDKN2A homozygous deletion), World Health Organization grade 4 (WHO grade 4) (male, age: 24 years) showing a growth pattern of gliomatosis cerebri with involvement of large parts of the right hemisphere with midline deviation (**a**), areas with low values for the apparent diffusion coefficient (ADC values) indicating high cellularity (**b**). Only focal contrast enhancement (**c**), corresponding high values for the relative cerebral blood volume (rCBV values) (**d**). **a** T2-weighted image, **b** ADC map, **c** T1-weighted image after i.v. application of contrast agent, colored rCBV map, respectively. Presence of a homozygous deletion of CDKN2A/B is a criterion for grading a diffusely infiltrating IDH-mutant astrocytoma as WHO tumor grade 4

The three entities within this group of tumors are the following: 1) astrocytoma, isocitrate dehydrogenase (IDH) mutant; 2) oligodendroglioma, IDH mutant and 1p/19q codeleted; 3) glioblastoma, IDH wildtype (Table 1; [1]). For *astrocytomas, IDH mutant*, a major new aspect since the last WHO classification of CNS tumors from 2016 is the introduction of a WHO tumor grade 4 beside the tumor grades 2 and 3 (Fig. 1). An astrocytoma, IDH mutant with WHO grade 4 is characterized by the presence of homozygous deletions of the tumor suppressor genes for CDKN2A or CDKN2B and/or microvascular proliferation and/or the presence of tumor necrosis [2–5]. Each of these three findings alone is sufficient for grading an IDH mutant diffuse astrocytoma as WHO tumor grade 4 [5]. CDKN2A/B are cell cycle regulatory proteins, and homozygous deletions of their genes alter their regulatory function leading to uncontrolled tumor cell proliferation [6]. This molecular alteration and even microvascular proliferation and tumor necrosis are absent in astrocytomas with WHO tumor grades 2 and 3. IDH mutant astrocytomas can occur in every region of the CNS but they most commonly are found in a supratentorial location, especially in the frontal lobe [4]. The average age is in the fourth decade and these gliomas only rarely occur in patients older than 55 years [7]. In a large series of 1360 adult-type diffuse astrocytomas with IDH mutation, gliomas with WHO grade 4 had a slightly higher mean age of 42 years compared to the average age of 38 years for tumors with WHO grades 2 and 3 [8]. Imaging reveals diffusely infiltrating tumors with a homogeneous mass in grade 2‑tumors and an increasing frequency of enhancement of contrast agent in tumors with higher tumor grades [4, 9]; however, it is important to know that contrast-enhancement is not a diagnostically reliable criterion to distinguish IDH mutant grades 2 and 3 gliomas from grade 4 gliomas. The IDH mutant gliomas are hypointense on T1-weighted sequences (T1-hypointense) and hyperintense on T2-weighted sequences (T2-hyperintense), whereas fluid attenuation inversion recovery MR sequences (FLAIR) often show a relative T2-hypointensity due to the relative suppression of the T2-signals, which are almost CSF isointense. This sign is called the T2/FLAIR-mismatch sign and is defined as the “presence of a complete or nearly complete hyperintense signal on T2-weighted MRI sequences, in combination with a relative hypointense signal on fluid attenuation inversion recovery (FLAIR) MR sequences except for a hyperintense peripheral rim” [10]. The T2/FLAIR-mismatch sign is highly specific for an IDH mutation, but sensitivity is comparably low, as it is present in approximately 50% of IDH mutant astrocytomas only [4, 11–13]. In IDH mutant astrocytomas WHO grade 4 with homozygous deletions of CDKN2A/B, histological findings, such as microvascular proliferation and tumor necrosis can be absent with the consequence that they radiologically show no contrast enhancement, no edema, and no necrosis and are therefore easily misinterpreted as low-grade gliomas. In these cases, WHO grade 4 can only be assigned after molecular examination. As additional important clinical information, integrated molecular analysis of 97 tumors showed that most IDH mutant astrocytomas grade 4 occurred de novo (68/97 cases), whereas 29/97 tumors emerged from a prior low-grade tumor [14].

**Table 1:** All entities of adult-type and pediatric type diffuse gliomas according to the new edition of the WHO classification of central nervous system tumors [1]: new aspects within the new edition, as well as important clinical, prognostic and molecular characteristics

**Adult-type diffuse gliomas**
*Astrocytoma, IDH-mutant:* introduction of the new tumor grade 4, characterized by homozygous deletions of the tumor suppressor genes for CDKN2A or CDKN2B and/or microvascular proliferation and/or the presence of tumor necrosis. By definition, these criteria are absent in astrocytomas, IDH-mutant, grades 2 and 3. Imaging signs of a high-grade tumor can be absent in grade 4 tumors, but clinical behavior is consistent with high malignancy [2–14]
*Oligodendroglioma, IDH-mutant and 1p/19q-codeleted:* imaging and histological criteria for diagnosis and grading are largely unchanged. Homozygous deletions of the *CDKN2A* gene can be present in oligodendrogliomas grade 3 and this finding has been confirmed to be a significant adverse prognostic factor [2, 15–22]
*Glioblastoma, IDH wildtype:* introduction of molecular criteria within the definition: presence of microvascular proliferation and/or necrosis and/or *EGFR* gene amplification and/or TERT promotor mutation and/or +7/−10 chromosome copy number changes. Imaging signs of a high-grade tumor can be absent, but tumors with presence of these molecular findings reveal the same poor outcome as for patients with glioblastomas showing typical morphological signs of malignancy [23–35]
**Pediatric-type diffuse low-grade gliomas**
*Diffuse astrocytoma, MYB or MYBL1 altered:* a new molecularly defined entity with tumor grade 1, most patients show initial epileptic seizures. Temporal lobe is the most frequent localization, but even cases within brainstem or cerebellum have been reported [36–40]
*Angiocentric glioma:* typical histology with bipolar tumor cells in perivascular spaces, *MCB/QKI* gene fusion is the typical molecular alteration. Similar imaging and clinical characteristics as the other low-grade entities of this group. Reports about adjacent focal cortical dysplasia and a stalk-like shape of the tumor outline projecting deeply towards the lateral ventricle in several cases [37, 41–48]
*Polymorphous low-grade neuroepithelial tumor of the young:* frequent calcifications and cystic lesions with otherwise similar imaging and clinical features compared with the other low-grade entities of this group. *BRAF V600E* mutation or even a gene fusion involving *FGFR2* or *FGFR3* as typical molecular alterations [49–58]
*Diffuse low-grade glioma, MAPK pathway-altered:* new entity with only few clinical and morphological data available up to now. Localization can be throughout the craniospinal axis but most cases occur within the hemispheres. Mutation of a MAPK pathway protein, mostly FGFR1 or BRAF V600E as the typical molecular alteration. Diagnosis should depend on exclusion of morphological features or a DNA methylation profile suggestive of an alternative tumor type [42, 59, 60]
**Pediatric-type diffuse high-grade gliomas**
*Diffuse midline glioma, H3K27-altered: *molecularly defined entity with H3K27M alteration due to mutation or other molecular events. Pathognomonic is a midline appearance including pons or other localizations like thalamus, hypothalamus, pineal region, cerebellum, and spinal cord. Considerable heterogeneity of histology and imaging ranging from exophytic growth with encasement of the basilar artery and contrast enhancement to sharply demarcated tumors without obvious imaging signs of malignancy. Prognosis is generally poor [61–79]
*Diffuse hemispheric glioma, H3G34-mutant:* a new molecularly defined entity with mutation of histone 3 gene at position 34. Prognosis is generally poor. Heterogeneity of histological and imaging findings with some tumors showing no contrast enhancement and no obvious signs of malignancy. Even these tumors, however, can be clearly recognized as high-grade tumors by means of FET-PET imaging [75, 80–84]
*Diffuse pediatric-type high-grade glioma, H3-wildtype and IDH-wildtype:* typical histological and imaging features of high-grade gliomas, but molecularly different to adult-type glioblastomas with IDH-wildtype (methylome analysis). Three molecularly and prognostically distinct subgroups (groups with frequent MYCN amplification, PDGFRA amplification, TERT mutation) with the group showing frequent MYCN amplification being associated with the shortest overall survival (median 14 months). Most tumors are localized within the hemispheres [85–91]
*Infant-type hemispheric glioma:* most prominent difference to the other entities within this group is the patients age limited to infancy. Typical genetic alterations are receptor tyrosine kinase fusions including the genes for the tyrosine kinases ROS1, ALK, MET and the NTRK family. Localization is superficial in the hemispheres with frequent cystic changes [92–94]

*ALK* anaplastic lymphoma receptor tyrosine kinase, *BRAF V600E* rapid accelerated fibrosarcoma oncogene type B, mutation at position 600 with exchange of valine by glutamic acid, *CDKN2A/B* cyclin dependent kinase inhibitor 2A/B, *EGFR* Epidermal growth factor receptor, *FET-PET* fluoroethyl-tyrosine positron emission tomography, *FGFR1–3* fibroblast growth factor receptor 1–3, *H3G34-mutation* mutation of the histone 3‑3A-gene with amino acid substitution at position 34 (G =  glycine), *H3K27-alteration* alteration of histone 3 at position 27 (K = lysine), *IDH* isocitrate dehydrogenase, *MAPK* mitogen-activated protein kinase, *MCB* Mlu‑I cell cycle box, *MET* mesenchymal epithelial transition proto-oncogene receptor tyrosine kinase, *MYB* V-Myb avian myeloblastosis viral oncogene homolog, *MYBL1* V-Myb avian myeloblastosis viral oncogene homolog-like 1, *NTKR1‑3* neurotrophic tyrosine kinase receptor 1–3, *PDGFRA* platelet-derived growth factor receptor A, *QKI* quaking homolog, KH domain RNA binding, *ROS1* V-Ros avian UR2 sarcoma virus oncogene homolog 1, *TERT* telomerase reverse transcriptase

For the diagnosis of an *oligodendroglioma*, the presence of both IDH mutation and 1p/19q codeletion are required for the diagnosis, which was already addressed in the last edition of the WHO classification from 2016 [2]. Of note, oligodendrogliomas display marked mutational heterogeneity with frequent occurrence of mutations of the genes for capicua transcriptional repressor (CIC) and telomerase reverse transcriptase (TERT) and even other mutations, but they are not sufficient for diagnosis [15]. Homozygous deletions of the CDKN2A gene have been described in a small subset of oligodendrogliomas grade 3 and this finding has been confirmed to be a significant adverse prognostic factor [6]. Beside this molecular alteration, which can serve as a molecular marker for oligodendrogliomas grade 3 in IDH-mutant and 1p/19q-codeleted oligodendrogliomas, pathological grading of oligodendrogliomas as WHO tumor grades 2 or 3 relies on the major histological criteria such as cytomorphology, cellularity and mitosis count [16, 17]. Patients with oligodendrogliomas have a median age in the fifth decade with WHO grade 3 tumors showing a slightly higher age of the patients, occurrence in children is a rare event. Most tumors are located in the frontal lobe and less frequently in the temporal or parietal lobe, only few tumors show a more caudal localization such as the brainstem [16, 17]. Oligodendrogliomas infiltrate the cortex and the cortical-subcortical junction; their outlines can appear as well-circumscribed masses, but also with indistinct tumor margins [16, 18]. Peritumoral edema is rare but can be seen in WHO grade 3 tumors [18–20]. Calcifications have been reported in oligodendrogliomas with WHO grades 2 and 3. In a small sample size of 17 cases, calcifications were only found in oligodendrogliomas grade 3 [19], but in another study with a large sample size of 45 cases with available calcification data, calcifications were also found in oligodendrogliomas with WHO grade 2. In this study, there was no significant association between the presence of calcifications and tumor grade [21]. High-grade oligodendrogliomas can also show focal necrosis [18, 19]. Contrast enhancement is present in both oligodendrogliomas with WHO grades 2 or 3, so that the enhancement should not be used as a diagnostic criterion for tumor grade 3. The enhancement of these gliomas is thought to be related to the typical vascular chicken wire-like configuration, which is a histological hallmark of oligodendrogliomas [18]. The average values for the apparent diffusion coefficient (ADC) have been shown to be significantly lower in high-grade oligodendrogliomas, but different studies reported a distinct overlap of ADC values between the tumor grades 2 and 3 [19, 22].

**Fig. 2:**
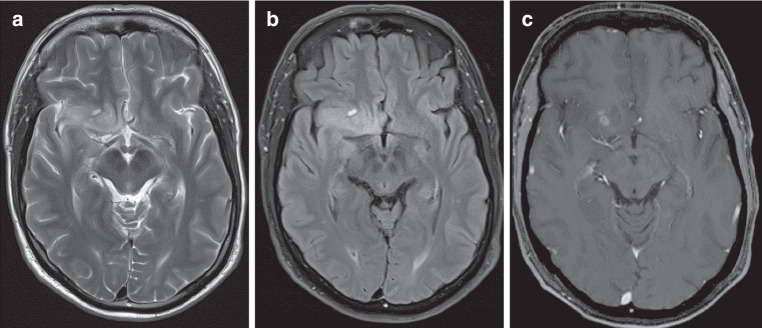
**a**–**c** Glioblastoma without mutation of isocitrate dehydrogenase (IDH-wildtype), with amplification of the gene for the epidermal growth factor receptor (EGFR amplification), gain of chromosome 7 and loss of chromosome 10, World Health Organization grade 4 (WHO grade 4) (male, age: 52 years) temporo-insular and frontobasal right with only small areas of contrast enhancement. **a** T2-weighted image, **b** fluid attenuation inversion recovery MR sequence (FLAIR), **c** T1-weighted image after i.v. application of contrast agent. Presence of EGFR amplification or telomerase reverse transcriptase promotor mutation (TERT promotor mutation) or chromosome 7+/10− copy number alterations or any combination of these features in a diffuse astrocytic glioma IDH-wildtype/H3-wildtype is diagnostic for a glioblastoma WHO grade 4, even if there is no obvious radiological sign of a glioblastoma such as necrosis

The major new aspect for the entity *glioblastoma, IDH-wildtype* is the introduction of molecular alterations as a part of its definition (Fig. 2). A diffuse astrocytic tumor without IDH mutation and without histone 3-alteration (H3 alteration) must be diagnosed as a glioblastoma, IDH wildtype with WHO tumor grade 4, if at least one of the following five histological and molecular features are present: microvascular proliferation and/or necrosis and/or EGFR gene amplification and/or TERT promotor mutation and/or +7/−10 chromosome copy number changes [23]. Based on this definition, it is possible that even tumors without typical histological or radiological signs of malignancy are glioblastomas, if at least one of the three molecular alterations mentioned above is present. It has been confirmed that these astrocytic tumors with molecular features of a glioblastoma reveal the same poor outcome as for those patients with glioblastomas showing typical morphological signs of malignancy. Also, a significantly shorter survival time compared to patients with IDH mutant astrocytomas has been confirmed [24, 25]. In a large database of 630 patients with glioblastomas, the age of the patients showed a range between 18 and 89 years, most patients (58%) were in the age group between 50 and 69 years [26]. Glioblastomas can occur throughout the neuroaxis, but most tumors are located within the cerebral hemispheres and can affect all lobes without preference, which is a major difference to IDH-mutant astrocytomas occurring preferentially in the frontal lobe [23, 27]. Another difference is the frequent occurrence of IDH-wildtype glioblastomas in multiple lobes and the frequent infiltration of the contralateral hemisphere through the corpus callosum [23, 27, 28]. Dominant imaging features of glioblastomas are diffuse infiltration of surrounding brain regions, spatial heterogeneity of signal intensities and contrast enhancement, often appearing as a typical ring-like enhancement around necrotic areas. Varying amounts of perifocal edema, necrosis, and even hemorrhages can be observed [23–28]. Beside standard sequences, susceptibility weighted imaging (SWI) and perfusion techniques (PWI), such as dynamic contrast enhanced (DCE) perfusion, as well as diffusion-weighting imaging (DWI) can improve the diagnostic accuracy. The SWI and PWI measure microbleeds and increased cerebral blood volume (rCBV) in the solid part of these tumors, and are thus radiological biomarkers for the neovascularization of glioblastomas. Low ADC values indicate the narrowed extracellular space in the solid part of glioblastomas due to the high density of proliferating tumor cells. Diffusion tensor-weighted imaging (DTI) and functional magnetic resonance imaging (fMRI) potentially enhance the set of available imaging methods, especially with respect to preoperative planning and treatment evaluation [29, 30]. Three important subtypes of the entity glioblastoma, IDH-wildtype, are giant cell glioblastomas, gliosarcomas, and epitheloid glioblastomas. A differentiation between these subtypes and a typical diffuse glioblastoma is not possible based on imaging characteristics in the individual case; however, there are several reports about the frequent occurrence of special radiological characteristics in these subtypes, such as a more circumscribed and less infiltrating margin in giant cell glioblastomas [31] and in epithelioid glioblastomas [32, 33]. For gliosarcomas, a frequent dural attachment and a dural tail sign have been reported, as well as infiltration of the adjacent bone and extracranial soft tissue in individual cases [34, 35].

## Pediatric-type Diffuse Low-grade Gliomas

Four entities are subsumed under the umbrella term of pediatric-type diffuse low-grade gliomas with each entity showing different histomorphological features in combination with distinct molecular alterations (Table 1). Of note, all these tumors are IDH-wildtype and even H3-wildtype. The first entity is the *diffuse astrocytoma, MYB or MYBL1 altered*, a low-grade tumor (WHO grade 1) with monomorphic glial cells and absent mitotic activity showing alterations of the genes for V-Myb avian myeloblastosis viral oncogene homolog (MYB) or V-Myb avian myeloblastosis viral oncogene homolog-like 1 (MYBL1) [36]. Although this tumor often shows well-defined borders in MRI, it may also show a more diffuse growth pattern [36, 37]. Even histologically, a diffuse growth into adjacent brain tissue is observed at least focally [38]. Therefore, recognition of a clear border between tumor and infiltrated tissue as well as the differentiation between neoplastic and non-neoplastic cells may be difficult [36–38]. In a large series of 26 cases, most patients (24/26) had epileptic seizures since childhood with a median age of 10 years during onset, median age during surgery was 29 years (range: 4–50 years) [38]. Most astrocytomas with MYB or MYBL1 alterations are localized within the temporal lobe, followed by the frontal and occipital lobe [36, 38]. Cerebral cortex is most frequently involved, but tumors can also be located within the cerebral white matter, the deep grey nuclei and, more rarely, within the brainstem and the cerebellum. Nearly all reported cases show no contrast enhancement, and microcystic structures with even larger cysts can be present. For cortical tumors, a lobulated appearance has been frequently reported, while tumors involving more caudal sites appeared more diffuse [37–40].

*Angiocentric gliomas* (WHO grade 1) are characterized by bipolar tumor cells cumulating within perivascular spaces, as well as by a typical molecular gene fusion between the gene for Mlu-I cell cycle box (*MCB* gene) and the gene for quaking homolog, KH domain RNA binding (*QKI* gene) [37, 41, 42]. Just like diffuse astrocytomas with MYB or MYBL1 alteration, angiocentric gliomas infiltrate adjacent brain structures diffusely, although they can appear as well-demarcated lesions in MRI [43]. As typical low-grade tumors, angiocentric gliomas do not show mitotic activity and a low Ki67 proliferation index [44]. Most patients present clinically with seizures and sometimes with headache and visual impairment [45]. Of note, a broad age range has been described (2–79 years) with an average age of 16 years at the time of initial diagnosis [45]. Tumors usually appear as a cortical or subcortical mass without contrast enhancement, calcifications and cystic structures can also be present. The most frequent localization is the temporal pole, followed by the frontal pole, parietal pole and brainstem [41, 45, 46]. In FLAIR sequences, a stalk-like shape of the tumor outline projecting deeply towards the lateral ventricle has been reported by several authors [45, 47, 48]. In a series of six angiocentric gliomas, histological and immunohistochemical investigations revealed a focal cortical dysplasia adjacent to the tumor in five cases, classified according to the International League Against Epilepsy (ILAE) as a dysplasia type IIIb (ILAE type IIIb) [44].

*Polymorphous low-grade neuroepithelial tumor of the young (PLNTY)* is also a WHO grade 1 tumor associated with seizures [49, 50]. The PLNTY is characterized by alterations of the mitogen-activated protein kinase-pathway (MAPK pathway) such as a mutation of the gene for the rapid accelerated fibrosarcoma oncogene type B at position 600 (BRAF V600E) or even a gene fusion involving the gene for the fibroblast growth factor receptor 2 or 3 (FGFR2 or FGFR3) in combination with typical histological features, such as oligodendroglioma-like areas, a diffuse growth pattern, as well as the expression of cluster of differentiation 34 (CD34) and oligodendrocyte transcription factor 2 (OLIG2) [50–53]. Despite the median age of 15.5 years [49], several patients with PLNTY up to the 6th decade of life have been reported [49, 54, 55]. Except for one tumor occurring in the third ventricle [52], all tumors described so far are supratentorial cortical or subcortical tumors with most cases localized within the temporal lobes [49, 54, 55]. A prominent radiological feature of many PLNTYs is a dense calcification appearing in CT and in T1 sequences. Even cystic lesions are often present. The tumor margin is well-delineated in most cases, a scarce contrast enhancement can be observed in a minority of cases [49, 56]. Two study groups reported the frequent occurrence of the so-called salt and pepper sign in T2-weighted and FLAIR sequences with alternating intensity throughout the tumor and it is claimed that this sign together with intense calcifications are specific imaging characteristics of PLNTY [50, 56]. For all tumors in the literature, a good outcome has been reported after total excision with only few cases showing evidence of local recurrence [50, 53, 56, 57]. As an exception, one tumor case of a 15-year-old girl has been reported with the unequivocal diagnosis of a PLNTY in the temporal lobe with FGFR3 gene fusion. A local recurrence appeared 17 months after near-total resection, showing the same molecular alteration after reoperation with additional molecular alterations typically seen in high-grade gliomas [58].

The *diffuse low-grade glioma, MAPK pathway-altered* is an entity with only few clinical and morphological data available up to now. The prognosis is better compared with IDH-mutant gliomas of WHO tumor grade 2, and a WHO tumor grade for this tumor type has yet to be assigned [59]. It is characterized by a mutation of a MAPK pathway protein, mostly FGFR1 or BRAF V600E. Histopathology seems to reveal some differences between these two mutations with frequent oligodendroglioma-like areas in FGFR1 mutated cases and more fibrillary glial tumor cells with OLIG2 positivity in BRAF V600E mutated cases [42, 59, 60]. In contrast to the three other entities of pediatric-type diffuse low-grade gliomas, this tumor type does not show a distinct single cluster within DNA methylation analysis and therefore, its diagnosis requires the exclusion of morphological features or a DNA methylation profile suggestive of an alternative tumor type. Localization of the tumor can be throughout the craniospinal axis with most tumors occurring in the cerebral hemispheres. It may appear as a heterogeneously enhancing mass with cystic changes [59].

## Pediatric-type Diffuse High-grade Gliomas

**Fig. 3:**
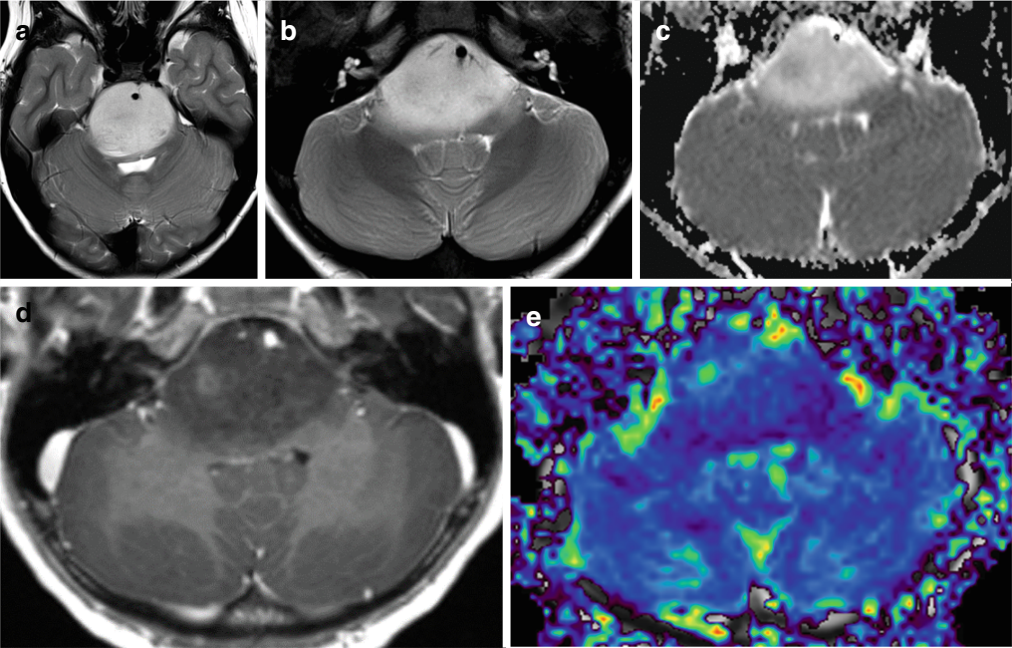
**a**–**e** Diffuse midline glioma with alteration of histone H3 at position 27 (H3K27-altered) (female, age: 7 years) with epicenter in the pons showing exophytic growth and encasement of the basilar artery (**a**, **b**). Focally low values for the apparent diffusion coefficient (ADC values) (**c**) indicate high cellularity, only focal contrast enhancement is present (**d**). High values for the relative cerebral blood volume (rCBV values) (**e**) indicate a pronounced vascularization. **a**–**b** T2-weighted images, **c** ADC map, **d** T1-weighted image after i.v. application of contrast agent, **e** colored rCBV map. Biopsy and molecular biological analysis revealed a H3K27M alteration. For diffuse gliomas located in the midline, an alteration of histone 3 at position 27 due to a mutation or due to other alterations such as enhancer of zeste homologs inhibitory protein-overexpression (EZHIP overexpression) or EGFR-mutation is diagnostic for a diffuse midline glioma, H3K27-altered

This group of tumors includes four different entities, the first entity is the *diffuse midline glioma (DMG), H3K27-altered*. The former term H3K27-mutant has been replaced by the term H3K27-altered, as instead of a mutation leading to an alteration of histone 3 at position 27 (H3K27-mutation), other molecular alterations such as an overexpression of the enhancer of zeste homologs inhibitory protein (EZHIP) can be present. Furthermore, a H3K27 alteration is no more considered specific for a DMG, as it has also been observed in some pilocytic astrocytomas, gangliogliomas and even in gliomas outside the midline [61–66]. A wide age range from 2 to 65 years and a median age of 11–14 years has been reported in different series [67, 68]. Prognosis of the patients is generally poor with a median overall survival of 1 year, whereas overall survival was significantly longer (6 years) for patients with H3-wildtype tumors [68]. Other factors significantly associated with a poorer prognosis are TP53 mutation and mutation of the histone H3.3 instead of H3.1 [69, 70]. Of note, longer survival times have been reported for individual patients older than 10 years and even for adult patients [70, 71]. Histopathology of H3K27-altered DMGs shows an astrocytic differentiation with infiltrative growth, but also a considerable range of other histological or cytological phenotypes can be present such as areas resembling primitive neuroectodermal tumors [67, 72]. Except for the above mentioned rare cases occurring outside the midline, a midline appearance of these tumors is almost pathognomonic including brainstem, thalamus unilateral or bilateral, hypothalamus, pineal region, cerebellum, and spinal cord [71, 73–75]. Imaging studies revealed a wide spectrum of radiological appearances reflecting the heterogeneity of the histopathology. Some tumors appear sharply demarcated or even without radiological signs of a high-grade tumor, other tumors can show a more diffuse growth pattern without a sharp demarcation of the tumor outline. Contrast enhancement can be distinct or even absent. Signal intensities of T1 and T2-weighted sequences can be considerably heterogeneous, hemorrhages and necrosis can be present [70–72, 74–77]. In pontine gliomas with H3K27M alteration, also termed diffuse intrinsic pons-glioma (DIPG), usually more than 50% of the pons surface is infiltrated in the axial plane [70]. A case series of 47 patients with diffuse midline gliomas with H3K27M alteration showed that patients with DIPGs are younger (median 7 years) compared to patients with gliomas of other locations such as thalamus (median 24 years) or spine (median 25 years) [67]. In DIPGs, the pons is often enlarged due to an exophytic growth with the basilar artery displaced and potentially engulfed (Fig. 3). Just as in the case shown in Fig. 3, a high T2-signal can be observed, also a weak or even absent contrast enhancement in T1-images at the time of diagnosis has been reported for many H3K27M-altered gliomas located in the pons ([76]; Fig. 3). Some DIPGs can show a gliomatosis cerebri-like pattern with diffuse leptomeningeal involvement and a histological pattern resembling a low-grade astrocytic lesion [72]. Of note, all these diffuse midline gliomas with H3K27M alteration behave as high-grade tumors irrespective of histological or imaging features [72, 74]. The need for a biopsy in such pontine tumors and for CNS tumors in children and adolescents in general is discussed according to the European Society for Paediatric Oncology (SIOPE) [72, 76–78]. A study by means of diffusion-weighted imaging comparing diffuse midline gliomas with and without H3 mutation revealed no significant differences regarding the ADC histogram parameters; however, significantly lower median ADC values were observed for those patients surviving less than 1 year after initial diagnosis [79].

**Fig. 4:**
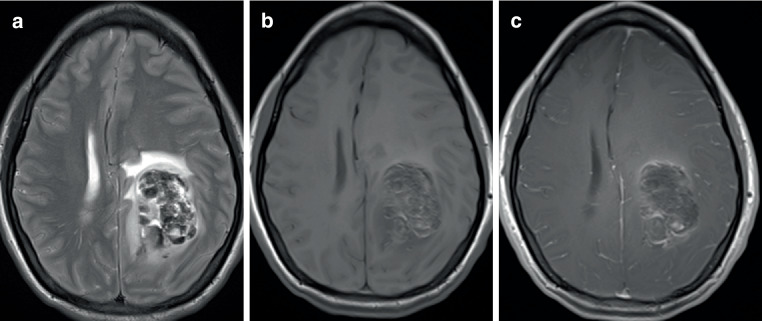
**a**–**c** Diffuse hemispheric glioma with mutation of the histone 3-3A gene at position 34 (H3G34-mutant) (female, age: 33 years) with involvement of the left hemisphere parietal showing heterogeneous signal intensities in T2-weighted and T1-weighted sequences (**a**, **b**) with hemorrhages and only faint contrast enhancement on T1-weighted image after i.v. application of contrast agent (**c**). **a** T2-weighted image, **b** T1-weighted image, **c** T1-weighted image after i.v. application of contrast agent. Molecular analysis revealed a H3G34-mutation of the H3.3 gene with amino acid substitution at position 34. This mutation is pathognomonic for a diffuse hemispheric glioma, H3G34-mutant

The second entity within this tumor group is the *diffuse hemispheric glioma, H3G34-mutant*. Just like diffuse midline gliomas with H3K27 alteration, diffuse hemispheric gliomas with mutation of the histone 3-3A gene at position 34 (H3G34-mutant) are not IDH-mutated. Histopathology is heterogeneous with some tumors showing predominantly features of a high-grade astrocytic tumor or even a glioblastoma, whereas in other cases a pattern resembling a primitive neuroectodermal tumor can be present [80, 81]. Prognosis of these tumors is generally poor. For different series, an overall median survival of 12 months [81] and 36 months [75] has been reported. In a large series of 81 cases, amplification of the platelet-derived growth factor receptor A (*PDGFRA*) oncogene had a negative statistical effect on survival time, whereas methylation of O6-methylguanine DNA methyltransferase (MGMT-methylation) had a favorable effect on the overall survival [80]. In contrast to H3K27-altered tumors occurring frequently in children, H3G34-mutated tumors primarily affect young adults with median ages of 18–26 years reported in different studies with an age range between 14 and 66 years [75, 81, 82]. Some tumors are monocentric, whereas others can show a gliomatosis-like multilobar involvement, main localizations are the parietal and the frontal lobe [75, 81–83]. Imaging findings can be considerably variable with some tumors showing infiltrative growth with leptomeningeal spread, necrosis, and distinct contrast enhancement. Even significant intratumoral bleeding can be present (Fig. 4). Other tumors show a slight or even absent contrast enhancement without obvious imaging features of a high-grade tumor [75, 81, 82, 84].

The *diffuse pediatric-type high-grade glioma, H3-wildtype and IDH-wildtype* shows typical histological features of high-grade gliomas grade 4 such as brisk mitotic activity, high proliferation index, as well as vascular proliferation and necrosis [85–87]. Age range of the tumors described so far is from early childhood to young adulthood, but research has focused very much on pediatric cases up to now and epidemiological data are still limited [85, 87]. These tumors can be differentiated from adult-type high-grade gliomas by DNA methylome analysis and additionally, this analysis reveals three molecularly and prognostically distinct subgroups: one subgroup is characterized by frequent occurrence of amplifications of the gene for V-Myc avian myelocytomatosis viral oncogene neuroblastoma derived homolog (MCYN amplification); another subgroup shows frequent PDGFRA amplifications and the last subgroup is characterized by frequent TERT promotor mutations or even EGFR amplifications. According to a large series of 74 pediatric patients, overall prognosis is poor with a median overall survival of 22 months. The three molecular groups differed significantly with respect to the median survival of the patients: time of survival was short for the group with frequent MYCN amplification (14 months) and longer for the groups with frequent PDGFRA amplification (21 months) and TERT mutation or EGFR amplification (44 months) [85]. The subgroup with frequent PDGFRA amplification reveals nearly the same spectrum of molecular alterations when compared with radiation-induced high-grade gliomas, which is an important finding for further understanding the mechanisms by which these secondary tumors arise [88, 89]. Most diffuse pediatric-type high-grade gliomas with H3 and IDH wildtypes are localized within the cerebral hemispheres, but even thalamic, sellar and infratentorial tumors within the brainstem have been reported [85, 86, 90]. Up to now, no specific differences regarding conventional imaging characteristics have been observed when comparing these tumors with other high-grade gliomas. Irrespective of their localization, most tumors show mass effects with T1 hypointensity, contrast enhancement, T2/FLAIR-hyperintensity of varying degree, indistinct margins and a perifocal edema [76, 91].

The fourth entity of pediatric-type diffuse high-grade gliomas is the *infant-type hemispheric glioma* (Table 1). Even these tumors show typical histomorphological high-grade features, such as high cellularity, palisading necrosis, and microvascular proliferation. In some tumors, also low-grade areas with less cellularity can be present [92]. Beside different molecular alterations compared with the other entities of pediatric type high-grade gliomas described previously, the prominent difference is the age distribution limited to infancy. One study on a large series reports an age of the patients limited to the first year of life with a median age of 2.8 months [93]. Another study provided evidence that this entity is indeed a tumor of infancy but not necessarily limited to the first year of life [92]. Tumors show a distinct cluster in DNA methylome analysis, typical genetic alterations are receptor tyrosine kinase fusions including the genes for the tyrosine kinases V-Ros avian UR2 sarcoma virus oncogene homolog 1 (ROS1), anaplastic lymphoma receptor tyrosine kinase (ALK), mesenchymal epithelial transition proto-oncogene receptor tyrosine kinase (MET) and the family of neurotrophic tyrosine kinase receptors (NTRK family) [92–94]. For tumor cases described so far, prognosis of children with infant-type hemispheric glioma seems to be better compared with the other pediatric-type high-grade gliomas with significant differences between the molecular alterations: For a group of 27 patients in total, 5‑year overall survival was 53.8%, 25.0% and 42.9% for patients with ALK, ROS1, and NTRK fusions of the tumors, respectively [93]. The need for further prospective evaluation of clinical information is addressed [92, 93]. An infant-type hemispheric glioma should be considered if an infant presents with a huge supratentorial mass, vasogenic edema and significant mass effect. The tumor is located superficially in the cerebral hemispheres, often involving the meninges. Necrosis can be present, as well as cystic formations, which can be considerably large. If present, these cystic structures sharply alternate with solid tumor areas [92–94].

## Discussion

The differentiation between diffuse gliomas of the adult type and the pediatric type is primarily based on a completely different spectrum of molecular alterations characterizing the three large groups of diffuse gliomas within the 5th edition of the WHO classification of CNS tumors (Table 1). Additionally, pediatric-type diffuse gliomas show clear cut molecular biological differences when comparing low-grade and high-grade tumors. Despite the designation pediatric type and adult type, however, it is clinically important to realize the overlapping age distribution of all diffuse gliomas, as pediatric-type diffuse gliomas can occur in adults, as well as adult-type diffuse gliomas can occur in children [95]. A second new major aspect in clinical diagnostics is the possibility that four entities with molecular features of diffuse high-grade gliomas may lack MR imaging features of a high-grade tumor. This applies to diffuse astrocytomas with IDH mutation showing a homozygous deletion of the *CDKN2A* or *CDKN2B* gene, and for glioblastomas with IDH wildtype showing at least one of three typical molecular alterations: *EGFR* gene amplification, TERT promotor mutation, +7/−10 chromosome copy number changes. Diffuse midline gliomas with H3K27 alteration and diffuse hemispheric gliomas with H3G34 mutation might also lack morphological signs of a higher tumor grade, but even these tumors will show typical alterations after molecular analysis. Of note, even a preoperative confirmation of malignancy in several diffuse hemispheric gliomas with H3G34 mutation but without imaging features of a high-grade tumor could be achieved by means of ^18^F‑FET-PET [84]. Especially for those diffuse gliomas without or with questionable features of a higher tumor grade, there is considerable motivation and discussion concerning the general need for a biopsy assessment [78, 96]. Several important differential diagnoses for diffuse high-grade gliomas must be addressed, especially from the group of circumscribed astrocytic gliomas. This applies to pleomorphic xanthoastrocytomas with individual cases showing contrast enhancement, indistinct margins and perifocal edema [97, 98]. Histological and molecular examination, however, will show a different immunophenotype and molecular profile when compared with diffuse astrocytic gliomas. Another important differential diagnosis is the high-grade astrocytoma with piloid features. This new entity can show a glioblastoma-like morphology, whereas other cases may show no obvious imaging features of a high-grade tumor [99, 100]. Final diagnosis of this tumor type will depend on DNA methylation profiling, as neither histopathology nor imaging or genetic analysis alone will provide a clear differentiation from other tumor types [100].

The four entities of pediatric-type diffuse low-grade gliomas show a considerable overlap of imaging characteristics. Most tumors occur within the hemispheres and cystic structures can be present. Even a focal or nodular contrast enhancement can be present in individual cases. Of major importance for routine diagnostic purposes is to be aware of the fact that these tumors have been observed in older patients within the 6th up to the 8th decade of life. An exception is the diffuse low-grade glioma, MAPK pathway-altered, as reports on this entity have focused on tumors in children up to now [59]. For differential diagnosis of diffuse low-grade gliomas, glioneuronal tumors must be considered. Imaging appearance of gangliogliomas may overlap with low-grade gliomas, but they usually show a higher rCBV without restriction on DWI. Cystic changes and calcification are often present [101]. Dysembryoplastic neuroepithelial tumor (DNT) is in general an important differential diagnosis for low-grade gliomas, but individual DNT cases have been described showing not the typical sharply defined margin but a more diffuse extension into the deeper brain structures with contrast enhancement. Therefore, DNT may also show overlapping imaging features when compared with diffuse high-grade gliomas [102]. Differentiation between pediatric-type diffuse low-grade and high-grade tumors can be challenging in the case of H3K27-altered or H3G34-mutated tumors showing no imaging signs of malignancy; however, the clear-cut differences between low-grade and high-grade gliomas concerning mutation analysis and methylation profiling provides a clear recognition of the individual tumor entity.

In summary, the article provided a detailed presentation of all important morphological, molecular, and clinical characteristics of adult-type and pediatric-type gliomas as a  basis for routine neuroradiological diagnostics. The fact that not every high-grade glioma will show imaging features of a higher tumor grade should be considered as a progress in molecular and clinical research: Many tumors diagnosed as low-grade tumors in former times can be recognized as high-grade tumors by means of their molecular profile with all consequences for further treatment. This opens the door for prospective studies on therapeutic approaches with small molecule inhibitors. There is the need for a further clinical and diagnostic assessment of the 11 tumor entities described in this review, and the neuroradiologist is called upon to contribute to the morphological characterization of larger tumor collectives.
